# Immuno-Spin Trapping-Based Detection of Oxidative Modifications in Cardiomyocytes and Coronary Endothelium in the Progression of Heart Failure in Tgαq*44 Mice

**DOI:** 10.3389/fimmu.2018.00938

**Published:** 2018-05-07

**Authors:** Bartosz Proniewski, Joanna Czarny, Tamara I. Khomich, Kamil Kus, Agnieszka Zakrzewska, Stefan Chlopicki

**Affiliations:** ^1^Jagiellonian Centre for Experimental Therapeutics (JCET), Jagiellonian University, Krakow, Poland; ^2^Institute of Pharmacology and Biochemistry, NAS of Belarus, Grodno, Belarus; ^3^Chair of Pharmacology, Jagiellonian University Medical College, Krakow, Poland

**Keywords:** immuno-spin trapping, oxidative stress, oxidative modifications, heart failure, cardiomyocytes, coronary endothelium, Tgαq*44 murine model, DMPO

## Abstract

Recent studies suggest both beneficial and detrimental role of increased reactive oxygen species and oxidative stress in heart failure (HF). However, it is not clear at which stage oxidative stress and oxidative modifications occur in the endothelium in relation to cardiomyocytes in non-ischemic HF. Furthermore, most methods used to date to study oxidative stress are either non-specific or require tissue homogenization. In this study, we used immuno-spin trapping (IST) technique with fluorescent microscopy-based detection of DMPO nitrone adducts to localize and quantify oxidative modifications of the hearts from Tgαq*44 mice; a murine model of HF driven by cardiomyocyte-specific overexpression of Gαq* protein. Tgαq*44 mice and age-matched FVB controls at early, transition, and late stages of HF progression were injected with DMPO *in vivo* and analyzed *ex vivo* for DMPO nitrone adducts signals. Progressive oxidative modifications in cardiomyocytes, as evidenced by the elevation of DMPO nitrone adducts, were detected in hearts from 10- to 16-month-old, but not in 8-month-old Tgαq*44 mice, as compared with age-matched FVB mice. The DMPO nitrone adducts were detected in left and right ventricle, septum, and papillary muscle. Surprisingly, significant elevation of DMPO nitrone adducts was also present in the coronary endothelium both in large arteries and in microcirculation simultaneously, as in cardiomyocytes, starting from 10-month-old Tgαq*44 mice. On the other hand, superoxide production in heart homogenates was elevated already in 6-month-old Tgαq*44 mice and progressively increased to high levels in 14-month-old Tgαq*44 mice, while the enzymatic activity of catalase, glutathione reductase, and glutathione peroxidase was all elevated as early as in 4-month-old Tgαq*44 mice and stayed at a similar level in 14-month-old Tgαq*44. In summary, this study demonstrates that IST represents a unique method that allows to quantify oxidative modifications in cardiomyocytes and coronary endothelium in the heart. In Tgαq*44 mice with slowly developing HF, driven by cardiomyocyte-specific overexpression of Gαq* protein, an increase in superoxide production, despite compensatory activation of antioxidative mechanisms, results in the development of oxidative modifications not only in cardiomyocytes but also in coronary endothelium, at the transition phase of HF, before the end-stage disease.

## Introduction

Cardiovascular diseases remain one of the most frequent causes of death globally, with an estimated 17.3 million deaths in 2013 and oxidative stress-related mechanisms play a dominant role in their pathogenesis including heart failure (HF) (1, 2). The heart is one of the most oxygen-consuming organs, and therefore changes in redox signaling play a crucial role in the pathophysiology of both acute and chronic diseases affecting the myocardium (3). In the cardiomyocytes, numerous sources of reactive oxygen species are identified, such as the mitochondria (4), NADPH oxidases (Noxs), uncoupled NO synthase, xanthine oxidase, and monoamine oxidase-A (5). Among seven Nox isoforms known to date, Nox2 and Nox4 are found in the cardiomyocytes (6). While Nox2 activation is responsible for increased superoxide production in the myocardium and promotes disease progression (7), the Nox4 isoform is reported to have either a beneficial (8), or detrimental role (9), depending on the studied model (10). Oxidative stress is linked with inflammation and fibrosis that play also an important role in HF progression (11–13). Progression of oxidative stress is prevented by various antioxidant mechanisms including endogenous antioxidant enzymes. Recently reductive stress has also been identified as an important factor in pathophysiology of oxidative stress in HF (14, 15).

Methods used to date to study oxidative stress rely on the detection of either steady-state intermediates or end products of oxidation or measurements of oxidative modifications of an exogenous probing molecule, such as lucigenin, [(3-boronophenyl)methyl]triphenyl-phosphonium, monobromide (mitoB) (16, 17), dihydroethidium (DHE), mitochondria-targeted hydroethidine (MitoSOX^®^), 10-acetyl-3,7-dihydroxyphenoxazine (*N*-acetyl-3,7-dihydroxyphenoxazine) (AmplexRed) or various EPR spin traps and probes (18–21). Depending on the choice of methodology, the aforementioned techniques have significant limitations, as they can either be specific to a predetermined oxidant [e.g., high performance liquid chromatography (HPLC) detection of DHE oxidation by superoxide (22)], eluding the information about the specific sites of oxidative stress due to required tissue homogenization, or allow localization of the oxidant generation at the expense of specificity [e.g., fluorescent detection of DHE oxidation (23)]. Some of these methodologies are also burdened with probe redox cycling (e.g., lucigenin), leading to possible over-estimation of oxidant abundance, or with high unspecific reactivity leading to high background and low sensitivity toward subtle changes. Moreover, these techniques leave an important question unanswered—what are the long-term repercussions in terms of tissue damage of a particular level of oxidative stress detected. Protein carbonylation, based on detection of dinitrophenyl hydrazine (24) or 3-nitrotyrosine levels using HPLC (25) are common methods to assess protein modifications. Other techniques allow for quantification of lipid peroxidation end products, such as 4-hydroxynonenal or malondialdehyde, which are commonly detected as their covalent protein adducts using Western Blot or immunohistochemistry with specific antibodies. Being a distinct marker of lipid peroxidation and an indication of increased oxidative stress, these assays are, however, more qualitative then quantitative as these are prone to variable levels of modifications, baseline variations, antibody specificity issues, and cross-reactivity (26). Oxidative modifications to DNA are most frequently studied with the assays for 8-hydroxy-2′-deoxyguanosine (27) to analyze oxidatively modified guanine (28). A number of other oxidative stress biomarkers/assays exist, e.g., thiobarbituric acid reactive substances (29, 30), oxidized low density lipoprotein or measurement of reduced glutathione (GSH)/oxidized glutathione (GSSG) ratio, reduced cysteine (Cys)/oxidized cysteine (CySS) ratio, or the non-enzymatic total antioxidant capacity assay (31). Their relevance to assess oxidant stress in HF has been extensively reviewed in literature (32–35).

A relatively new approach to study oxidative stress that can provide an additive insight is called immuno-spin trapping (IST) and was originally developed by Mason (36). It capitalizes on the specific, high reactivity of 5,5-dimethyl-1-pyrroline *N*-oxide (DMPO), an intracellular EPR spin trap, exhibiting low toxicity in cells (37) and animals (38) with damaged DNA in the nuclei and mitochondria as well as with protein, and lipid radicals (39–41). The covalent bond forms stable nitrone adducts upon DMPO injection *in vivo*, which can be then detected with DMPO-specific antibodies *ex vivo* (42) using immunohistochemistry or Western Blots (40, 43, 44), or alternatively it can be also used as a contrast agent in molecular MRI (45–49).

In this work, we have used IST with fluorescent detection of DMPO nitrone adducts to proteins and/or lipids to characterize and quantify the progression of oxidative modifications in the cardiomyocytes and coronary endothelium in hearts of Tgαq*44 mice with cardiomyocyte-specific overexpression of the Gαq* protein (50) mimicking constant neurohormonal overstimulation of cardiomyocytes by renin–angiotensin–aldosterone, sympathetic, and ET-1-dependent systems, mediated *via* angiotensin AT1, adrenergic α1, endothelin ET-A receptor stimulation, respectively. Tgαq*44 mice represent a unique and relevant model of human HF pathophysiology, on a molecular, morphological and functional level. Importantly, Tgαq*44 mice model is characterized by a prolonged course of HF progression with early activation of hypertrophic genes [atrial natriuretic peptide (ANP), brain natriuretic peptide (BNP), and myosin heavy chain beta (MHC-β)], cardiomyocyte hypertrophy, fibrosis (50, 51), and relatively long-term survival (52). The involvement of renin–angiotensin system (53), changes in ACE/ACE2 balance (54), metabolic remodeling (55), mitochondrial alterations (4), or coronary endothelial dysfunction (56) has been previously described in this model. Taking advantage of the protracted time course of progression to overt HF in Tgαq*44 mice, we have recently comprehensively analyzed the deterioration of cardiac function by MRI *in vivo*, identifying three distinct phases of HF progression reflecting early, transition and end-stage phases of HF in this model: initial alterations in cardiac performance including changes in LV strains and rotation, suggestive of diastolic dysfunction that coincides with impairment in atrial function (6 months of age); the transition phase, encompassing progressive impairment in basal systolic and diastolic cardiac performance, with preserved response to dobutamine (8–10 months of age); end-stage phase of HF with fully impaired systolic and diastolic cardiac performance, impaired response to dobutamine (54), and profoundly impaired physical activity (starting at the age of 12 months) (57). Overexpression of the Gαq* protein is limited to cardiomyocytes and results in increased superoxide generation in hearts of Tgαq*44 mice as well as coronary endothelial dysfunction in the end stage of HF (56). However, it is not known at which stage of HF development oxidative modifications occur in cardiomyocytes and coronary endothelium, and what is the temporal relationship for oxidative modifications in the cardiomyocytes as compared with coronary endothelium.

Accordingly, in this work, we used IST method to quantify oxidative modifications in the cardiomyocytes and coronary endothelium in the hearts of Tgαq*44 mice, HPLC-based DHE detection to quantify superoxide production in the heart and classical methods for the assessment of activities of cardiac antioxidant enzymes: superoxide dismutase (SOD), catalase (CAT), glutathione reductase (GR), and glutathione peroxidase (GPx). Tgαq*44 mice have been studied at age groups representative for three stages of HF progression: early, transition, and end-stage (3–6, 8–10, and 12–16 months of age, respectively) and compared with age-matched FVB mice. Our approach allowed for the investigation of onset and development of oxidative modifications in the cardiomyocytes as well as coronary endothelium of large and small vessels along the progression of HF, in relationship with increased superoxide production and activity of antioxidative mechanisms in the heart.

## Materials and Methods

### Animals

Transgenic, homozygous Tgαq*44 mice, characterized by cardiac-specific expression of activated Gαq protein, developed previously (50), as well as wild-type control mice (FVB) were bred at the Animal House of the Institute of Experimental and Clinical Medicine of the Polish Academy of Sciences in Warsaw. Successful transgene incorporation in hearts of Tgαq*44 mice was confirmed by PCR with transgene-specific primers. Increased mRNA level and protein expression of activated Gαq subunit in Tgαq*44 hearts were verified by RT-PCR and Western Blotting methods, respectively (50). Before the experiments, the animals were transported to the animal house at the Faculty of Pharmacy, Medical College, Jagiellonian University in Krakow (Poland). Mice were housed four to six per cage and maintained at 22–24°C under a 12-h light/day cycle with *ad libitum* access to water and rodent chow. Female Tgαq*44 mice of various ages were used: 3-month-old mice, *N* = 5 (for DHE analysis); 4-month-old mice, *N* = 6 (for antioxidant activity); 6-month-old mice, *N* = 13 (6 for DHE analysis and 7 for LC/MS–MS); 8-month-old mice, *N* = 5 (for IST); 9-month-old mice; *N* = 4 (for DHE analysis); 10-month-old mice, *N* = 5 (for IST); 12-month-old mice, *N* = 21 (5 for IST, 6 for antioxidant activity, and 10 for LC/MS–MS); 14-month-old mice, *N* = 20 (6 for IST, 8 for DHE analysis, and 6 for antioxidant activity), and 16-month-old mice, *N* = 4 (for IST). Age-matched FVB wild-type mice were used for comparison: 3-month-old mice, *N* = 5 (for DHE analysis); 4-month-old mice, *N* = 6 (for antioxidant activity); 6-month-old mice, *N* = 14 (6 for DHE analysis and 8 for LC/MS–MS); 8-month-old mice, *N* = 5 (for IST); 9-months-old mice; *N* = 4 (for DHE analysis); 10-month-old mice, *N* = 5 (for IST); 12-month-old mice, *N* = 16 (5 for IST, 4 for antioxidant activity, and 7 for LC/MS–MS); 14-month-old mice, *N* = 23 (6 for IST, 11 for DHE analysis, and 6 for antioxidant activity), and 16-month-old mice, *N* = 4 (for IST). All experimental procedures were compliant with the Guide for the Care and Use of Laboratory Animals published by the U.S. National Institutes of Health (NIH Publication No. 85-23, revised 1996) and were approved by the Second Local Ethical Committee on Animal Testing at the Institute of Pharmacology PAN in Krakow, Poland (permit no. 15/2016).

### Quantification of Oxidative Modifications by IST

#### DMPO Injection Protocol

A total dose of 1.5 g/kg DMPO was used, delivered in 3 equal intra peritoneal (i.p.) injections at approximately 24, 12, and 6 h before sacrifice (43). Body weight of all animals was measured just before the initial DMPO injection. Mice were sacrificed at the age of 8–16 months (ketamine and xylazine, 100 and 10 mg kg^−1^, respectively). The mouse chest was surgically opened and perfused *via* left (systemic circulation) and right (pulmonary circulation) ventricles with ice-cold PBS for total of 10 min. Hearts were isolated and immediately placed in ice-cold 30 mM KCl (dissolved in PBS) to ensure cardiac arrest in diastole. From each heart, the apex was cutoff and retained for Western Blot analysis. The remainder of the hearts was fixed in formalin and paraffin embedded.

#### Immunohistochemical Analysis of DMPO Nitrone Adducts in Cardiomyocytes

Formalin-fixed and paraffin-embedded hearts were cut into 5 µm slices on Accu-Cut SRM 200 (Sakura) rotational microtome. Antigen retrieval was performed according to the standard protocol using Leica Autostainer XL (Leica Biosystems). To visualize the extent of DMPO nitrone adducts, the slices were incubated with the primary anti-DMPO nitrone adduct antibody (1 h, dilution 1:300; Abcam, ab23702), secondary goat anti-rabbit IgG Cy3 (30 min, dilution 1:1,000; Jackson ImmunoResearch, cat no. 111-165-003) and Hoechst 33258 to visualize the nucleus (10 min, dilution 1:2,000; Sigma, cat no. 861405-100MG). Some slides were co-stained with biotinylated lectin to stain the endothelium (1 h, dilution 1:200; Vector Laboratories, cat no. B-1105) and visualized with Alexa Fluor^®^ 488 streptavidin (1 h, dilution 1:375; Jackson ImmunoResearch, cat no. 016-540-084). Slides were kept in the dark at 4°C until imaged. Slices without the primary anti-DMPO antibody served as blank control. For every animal *n* = 2 heart slices were analyzed. Randomly chosen homogenous, non-obstructed images covering the papillary muscle (*n* = 1/slice), right and left ventricle (*n* = 3/slice each), and the septum (*n* = 3/slice) were acquired with Axio Observer D1 (Zeiss) inverted microscope equipped with AxioCamHR3 camera and LD Plan-Neofluar 40×/0.6 Korr M27 objective in three channels: Hoechst (nucleus; excitation at 358 nm, emission at 461 nm), FITC (autoflourescence/endothelium; excitation at 494 nm, emission at 519 nm), and Cy3 (DMPO nitrone adducts; excitation at 552 nm, emission at 570 nm). Since blood vessels cover only few percent of imaged area (FVB mice: median = 2.8%, Q1 = 1.9% and Q3 = 4.6%; Tgαq*44: median = 3.1% Q1 = 1.9% and Q3 = 5.1%) the DMPO-specific fluorescent signal measured and analyzed in the whole cross-sections of the heart was considered to originate mainly from cardiomyocytes, thus is described thereafter to evaluate oxidative modifications in cardiac myocytes. Due to the broad age range of mice in this study, Tgαq*44 mice were analyzed with regards to their aged-matched FVB controls, as mice at the specific age were sacrificed on a single day. Images were analyzed in Columbus (PerkinElmer Inc.), and the DMPO nitrone adducts were expressed as the mean Cy3 fluorescence intensity, normalized to tissue autoflourescence (FITC channel), to overcome the bleaching effect. Furthermore, the heart specimens have been submerged in formalin for a different amount of time up to 4 months (for the oldest group), before the entire staining procedure for all groups has been done within a short period of time. For this reason, results from Tgαq*44 mice were first normalized to appropriate aged-matched controls, to become independent of any effects of various length of tissue pre-processing.

#### Immunohistochemical Analysis of DMPO Nitrone Adducts in Coronary Endothelium

Some slides were co-stained with biotinylated lectin to stain the endothelium (1 h, dilution 1:200; Vector Laboratories, cat no. B-1105) and visualized with Alexa Fluor^®^ 488 Streptavidin (1 h, dilution 1:375; Jackson ImmunoResearch, cat no. 016-540-084). Images were used to segment and quantify DMPO nitrone adducts within the coronary endothelium of large- and microvessels in ImageJ (58). For large coronaries, at least *n* = 6 vessels per mouse within the left ventricle were captured and segmented according to the following scheme: selection of the entire vessel and discerption of the remainder of the image, segmentation of the endothelial layer, based on Otsu threshold in the FITC channel, copy this selection onto the Cy3 channel, and measurement of the mean fluorescent signal. Results of endothelium-specific DMPO nitrone adducts are expressed as the mean Cy3 signal, normalized to the tissue autoflourescence and endothelial area. Detection of DMPO nitrone adducts in the coronary microvasculature of the left ventricle and septum (*n* = 47–72 images per age group) followed a similar analysis, and are expressed as the mean Cy3 signal within the lectin-positive area, normalized to tissue autoflourescence and to the number of microvessels per square millimeter, quantified using automated morphological manipulations in ImageJ software (i.e., conversion to binary, dilation, hole filling, erosion, and particle analysis) of images on the thresholded (“Otsu dark” Auto Threshold) FITC channel.

#### Western Blot Detection of DMPO Nitrone Adducts in Heart Apex Homogenates

Heart apex were weighed and homogenized in the Tissue Protein Extraction buffer (T-PER^®^, Thermo Fisher Scientific cat no. 78510) for protein extraction with protease and phosphatase inhibitors (Roche, cat no. 04693132001 and 04906837001). Protein concentration was measured with BCA assay. After addition of loading buffer, samples were heated at 95°C for 5 min and then frozen at −80°C. Samples were reduced and denatured by tris(2-carboxyethyl)phosphine (50 mM) instead of β-mercaptoethanol, as described previously by Khoo et al. (43). Each time, 30 µg of protein was loaded and run on the gel (Bio-Rad, cat no. 161-0185), then transferred to nitrocellulose membrane, blocked with 5% dry milk in TBST, and incubated overnight at 4°C with the primary DMPO nitrone adduct antibody (Abcam, ab23702). The appropriate HRP-conjugated secondary antibodies were from Santa Cruz Biotechnology (cat no. sc-2004), incubated for 1 h at room temperature. Equal protein loading was controlled after electrophoresis and transfer for gels and membranes, respectively, using stain-free technique provided by Bio-Rad (59). Blots were developed using enhanced chemiluminescence substrate (Bio-Rad, cat no. 1705061). Band intensity was assessed using Image Lab software.

### DHE-Based Analysis of Superoxide Production in the Heart

Mice aged at 3, 6, 9, and 14 months were used to assess superoxide production in the myocardium using HPLC detection of 2-hydroxyethiudium (2-OH-E+). Subsequent to anesthesia (100 mg kg^−1^ ketamine + 10 mg kg^−1^ xylazine, i.p.), the mouse chest was surgically opened and perfused *via* left (systemic circulation) and right (pulmonary circulation) ventricles with ice-cold PBS for a total of 10 min. Hearts were isolated, sectioned into appropriate fragments when necessary and incubated at 37°C for 45 min in freshly prepared 500 µl of 10 µM DHE in PBS under low light conditions. The tissues were dried on a piece of Kimwipe paper, snap frozen in liquid nitrogen, and stored at −80°C. On the day of HPLC analysis, the samples were thawed on ice, homogenized in 500 µl in 0.1% Triton X–100 (dissolved in PBS) and centrifuged (at 1,000 *g* for 5 min at 4°C). 100 µl of the supernatant was collected, mixed 1:1 (v/v) with 0.2 M HClO_4_ in MeOH, vortexed for 10 s and, kept on ice for 90 min. Next, the samples were centrifuged at 16,600 *g* for 30 min at 4°C, 120 µl of the resulting supernatant was collected and mixed 1:1 (v/v) with 1 M KPi pH 2.6 and centrifuged once more at 16,600 *g* for 15 min at 4°C. 200 µl of this homogenate was used for HPLC analysis of the DHE oxidation products, as described previously (60) with minor modifications (61). Results were normalized to the protein content of each sample, assessed in the initial supernatant.

### Assessment of Endogenous Antioxidant Systems and Redox State in the Heart

Heart tissue homogenate supernatant was used to estimate the levels of endogenous antioxidants in 4-, 12-, and 14-month-old mice by measuring activities of SOD, CAT, GPx, GR, as well as reduced GSH. After sacrifice, the hearts were quickly removed, washed out in cold 0.9% NaCl, dried on the filter, and placed into liquid nitrogen for freezing. The tissues were kept in −80°C. The homogenates from frozen tissues were prepared in glass homogenizer using ice-cold (4°C) 0.01 M PBS buffer, pH 7.2, containing 0.15 M KCl in dilution (w/v) 1:9. Homogenates were centrifuged at 13,000 rpm for 15 min (4°C), and supernatant was taken for analysis.

#### Assay of SOD Activity

The total superoxide dismutase (SOD) activity was determined according to the method of Misra and Fridovich (62) at 30°C. Supernatant (10 µl) was added to 960 µl of carbonate buffer (0.05 M, pH 10.2, 0.1 mM EDTA). Then epinephrine 30 mM (30 µl) (in 0.05% acetic acid) was added, and absorbance was measured at 480 nm for 4 min on a PerkinElmer Lambda 950 spectrophotometer. SOD activity was expressed in unit per milligram protein. Amount of enzyme that inhibits the oxidation of epinephrine by 50% was defined as 1 U.

#### Assay of Catalase Activity

The method of Aebi (63) was used to measure the catalase activity. In brief, to a quartz cuvette, 50 µl of supernatant was added to 650 µl of 50 mM potassium phosphate buffer, and the reaction was started by addition of 300 µl of 30 mM hydrogen peroxide (H_2_O_2_). The decomposition of H_2_O_2_ was monitored at 240 nm, 30°C for 3 min. The catalase activity was expressed as micromoles of H_2_O_2_ consumed per minute per milligrams of sample protein.

#### Assay of GR Activity

Glutathione reductase (GR) activity was determined according to method of Carlberg and Mannervik (64). NADPH (50 µl; 2 mM) in 10 mM Tris buffer (pH 7.0) was added in a cuvette containing 50 µl of GSSG (20 mM) in phosphate buffer (0.5 M, pH 7.0, 0.1 mM EDTA) and 850 µl of phosphate buffer. Supernatant (50 µl) was added to the NADPH–GSSG-buffered solution, and absorbance was measured at 340 nm for 3 min at 37°C. The molar extinction coefficient of 6.22 × 10^3^ M cm^−1^ was used to determine GR activity. One unit of activity was equal to the millimolars of NADPH oxidized per minute per milligrams of protein.

#### Assay of GPx Activity

The modified method of V. Moin was used to determine activity of Glutathione Peroxidase (GPx) (65). The optimal conditions for assays of enzyme activity were as follows: the incubation medium consisted of 0.1 M Tris–HCl buffer, pH 8.5 containing 5 mM EDTA; 10 mM sodium azide; 4.0 mM reduced glutathione; and 1.4 mM tert-butyl hydroperoxide. Supernatant (10–50 µl) was added to the mixture and after 5 min of incubation at 37°C Ellman’s reagent was added. The concentration of reduced glutathione before and after incubation was determined colorimetrically using standard Ellman’s reaction (see below) in control and tested samples.

#### Reduced Glutathione

Reduced glutathione was estimated by the method of Ellman (66). The reaction mixture consisted of 10% trichloroacetic acid, 0.1 mM 5,5′-dithio-bis (2-nitrobenzoic acid) in 0.1 M phosphate buffer (pH 8.0) and requisite amount of tissue supernatant. Absorbance was measured at 412 nm.

Protein contents in samples were determined by the method of Bradford (67) with BSA as the standard.

#### Measurement of GSH/GSSG and NADPH/NADP Ratio

To quantify the GSH/GSSG and the NADPH/NADP ratios in whole-heart homogenates from 6- and 12-month-old Tgαq*44 and FVB mice, LC/MS–MS-based method was used as described previously (68). Briefly, the heart tissue samples were homogenized in PBS containing BHT (1:6 w/v). An aliquot of 10 µl of plasma or tissue homogenate was used to extract the metabolites by addition of 0.5 ml of dry-ice-cold (−70°C) extraction mixture (acetonitrile:methanol:water 5:2:3, v/v/v). The extraction mixture was prepared at least 4–5 h before the experiment and placed in freezer. The samples were vortexed for 5 min and placed on dry ice for 30 min for protein precipitation. After that time, samples were centrifuged at 15,000 *g*, 4°C for 15 min. Supernatant was lyophilized, and dry extract were kept at −80°C until analysis. The metabolite extracts were reconstituted in 50 µl of LC/MS–MS grade water and was injected onto LC/MS–MS column. Chromatographic studies were performed on a UFLC Nexera (Shimadzu, Kyoto, Japan). The analytical column employed was an Acquity UPLC BEH C18, 1.7 µm 2.1 mm × 100 mm (Waters, Milford, MA, USA). The samples were measured twice, injecting onto analytical column 5 µl of sample acetonitrile:100 mM ammonium formate (pH 5.0) 95:5 v/v and 5 mM ammonium formate (pH 5.0) were used as a mobile phase in gradient elution in a run time of 8 min for positive and 5.5 min for negative ionization. Detection was performed with a QTRAP 5500 mass spectrometer (Sciex, Framingham, MA, USA) employed with an electrospray interface operated in positive and negative ionization MRM modes. The ion source operation conditions were as follows: curtain gas: 25 psi, collision gas: medium, temperature: 500°C, ion source gas 1: 40 arb., ion source gas 2: 50 arb., and ion spray voltage: 5,500 V and −4,500 V for positive and negative ionization modes, respectively.

### Statistical Analysis

Data are expressed as median and interquartile ranges (Q1–Q3, IQR). Normality of the data distribution was tested with Shapiro–Wilk’s test and variance homogeneity using Bartlett’s or *F* test. The significance of differences between age-matched groups was analyzed with two-sided Student’s *t*-test or the non-parametric Mann–Whitney *U* test. Differences along the progression of HF were analyzed using one-way ANOVA followed by *post hoc* multiple comparisons LSD Fisher’s test or Kruskal–Wallis non-parametric test, followed by *post hoc* multiple comparisons Dunn’s test, depending on the variable distribution. Detailed descriptions can be found in the caption under each figure. Statistical tests were done using GraphPad Prism 7 (GraphPad Software, Inc., CA, USA) software. *P* values < 0.05 were considered statistically significant.

## Results

### Oxidative Modifications in Cardiomyocytes

**Figure 1:**
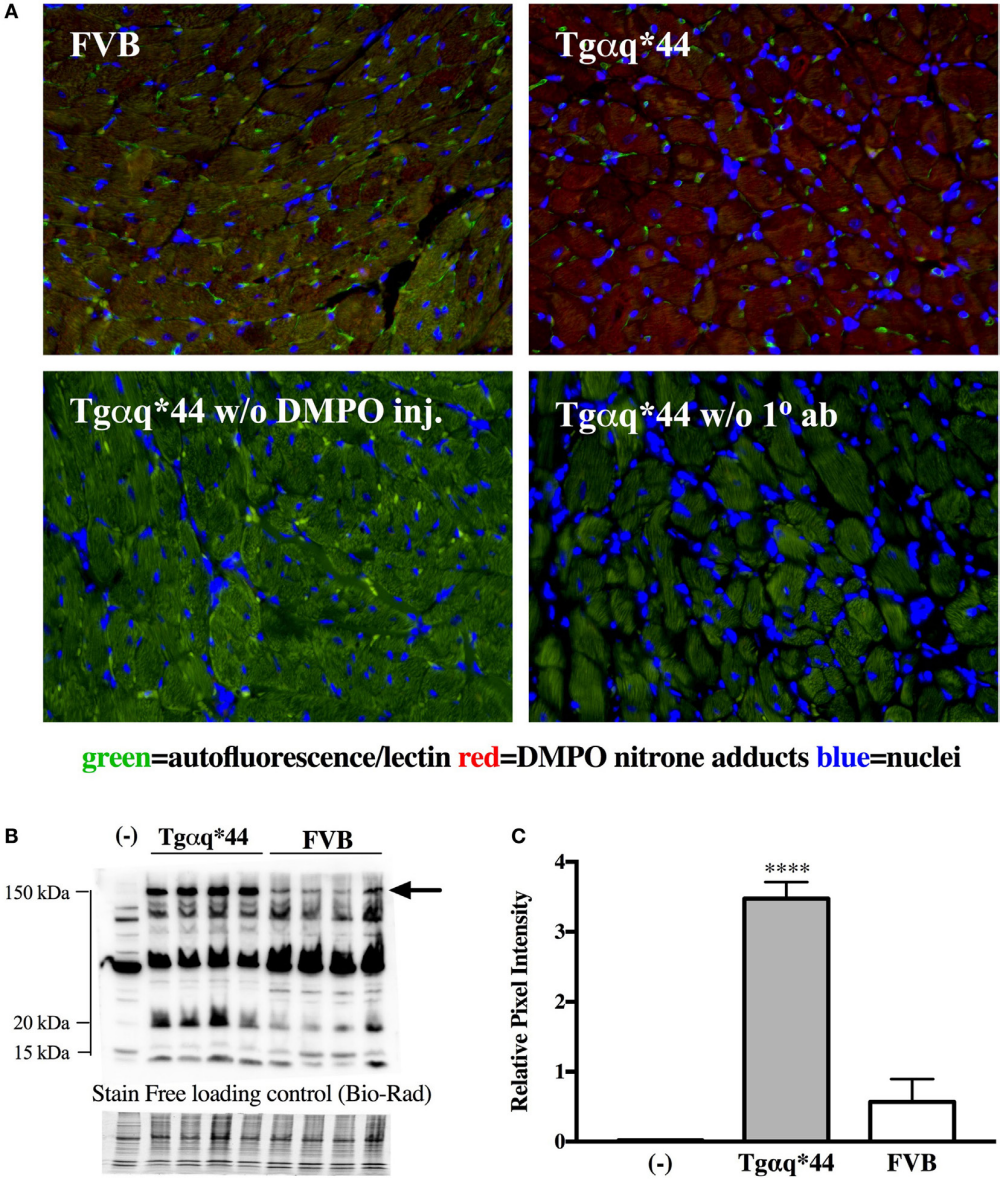
DMPO immuno-spin trapping in the myocardium of Tgαq*44 vs FVB mice. **(A)** Immunohistochemical images of hearts from FVB and Tgαq*44 mice, along with methodological negative controls without DMPO injection (w/o DMPO inj.) or without anti-DMPO primary Ab (w/o 1° ab) (see Materials and Methods for details). **(B)** Representative DMPO nitrone adduct Western Blots of heart apex from 16-month-old mice. Equal protein loading was controlled after electrophoresis and transfer for gels and membranes, respectively, using a stain-free technique provided by Bio-Rad and is shown below the Western Blot image (see Section “Materials and Methods” for details). **(C)** Densitometric analysis of the 150 kDa DMPO nitrone adduct-specific lane, for Western Blots marked in panel **(B)** with an arrow, presented as median ± IQR. For panels **(B,C)**, (-) indicates tissue from age-matched FVB mice injected with 0.9% NaCl. Statistical significance was assessed using an unpaired two-tailed *t*-test, ****P < 0.001.

**Figure 2:**
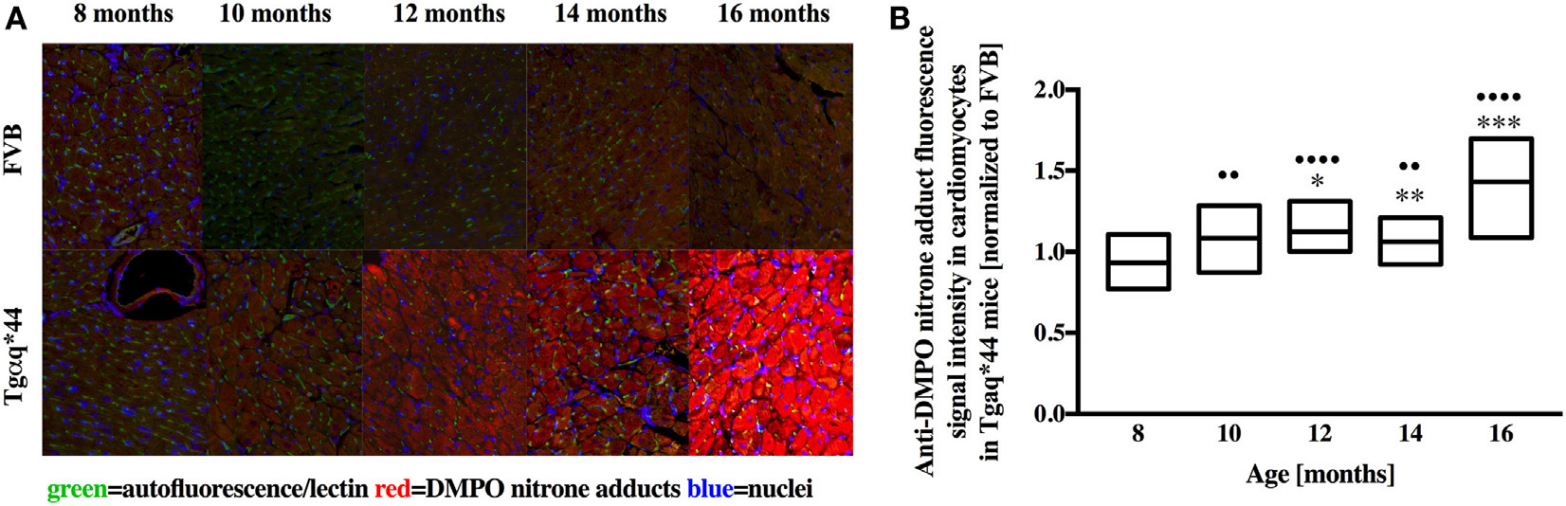
Immunohistochemical detection of DMPO nitrone adducts in the hearts of Tgαq*44 normalized to aged-matched FVB mice. **(A)** Representative images of the left ventricle from 8- to 16-month-old mice. **(B)** Quantification of oxidative modifications, based on the DMPO fluorescent signal, normalized to aged-matched FVB mice (see Section “Materials and Methods” for details). Results presented as median ± IQR. Statistical significance was assessed using the Kruskal–Wallis ANOVA, with *post hoc* Dunn’s test to compare oxidative modifications to 8-month-old group, with ^•^•^^*P* < 0.01 and ^••••^*P* < 0.001. Statistical significance between Tgαq*44 and FVB mice in aged-matched groups was assessed using non-parametric, two-sided Mann–Whitney *U* test, *P < 0.05, **P < 0.01, and ***P < 0.005. *N* = 4–6 mice per group, *n* = 10 slices per mice.

**Figure 3:**
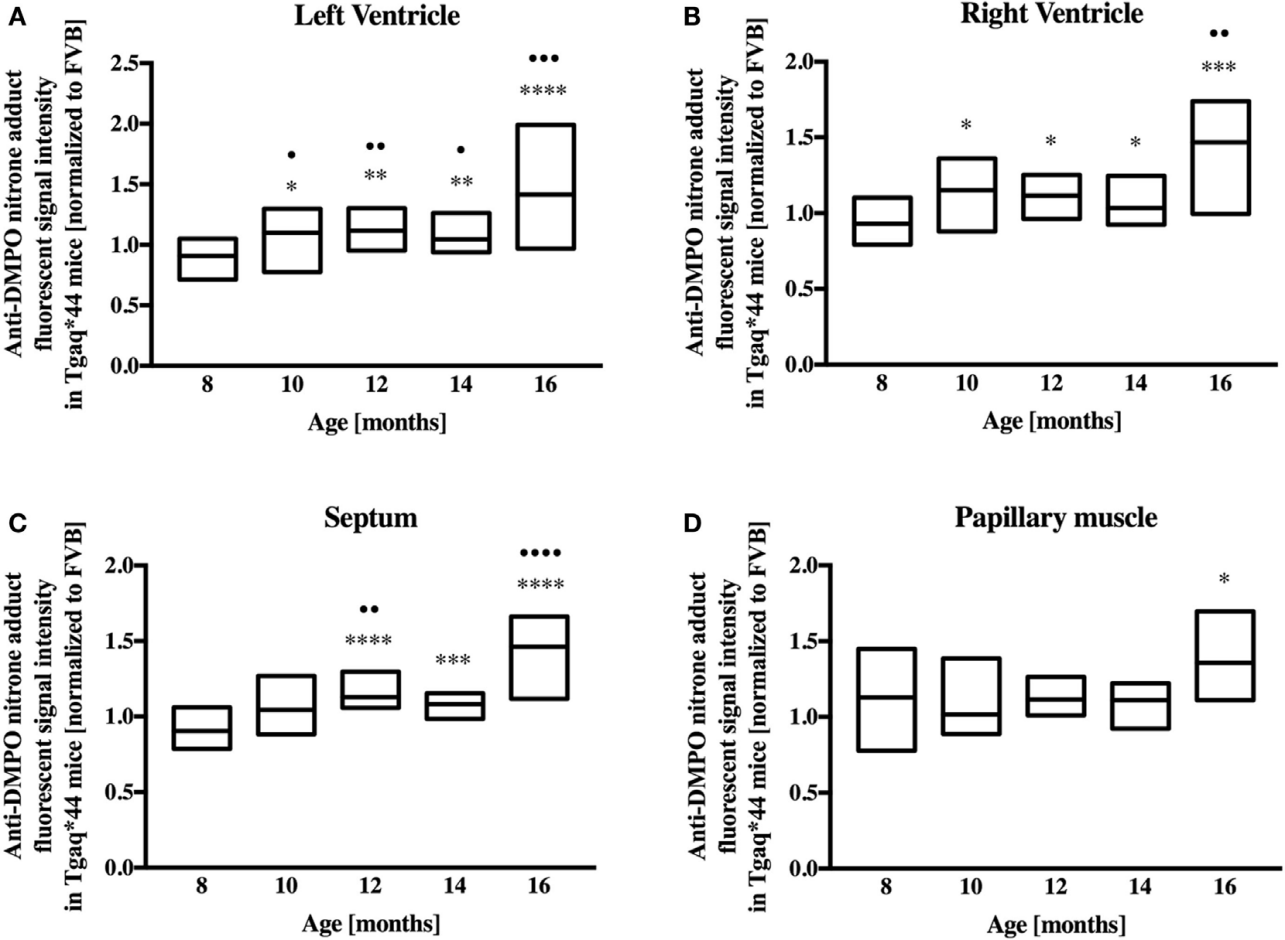
Regional oxidative modifications analyzed using immunohistochemical detection of DMPO nitrone adducts in hearts of Tgαq*44 normalized to aged-matched FVB mice. Quantification of oxidative modifications in the **(A)** left and **(B)** right ventricles, **(C)** septum, and **(D)** papillary muscle of the myocardium showed inhomogeneous distribution of oxidative modifications in the analyzed regions. Results presented as median ± IQR. Statistical significance of changes compared with the 8-month-old group for the left ventricle, right ventricle, and septum were assessed using Kruskal–Wallis ANOVA with *post hoc* Dunn’s test (*n* = 24–30 fields per group), while data for the papillary muscle were analyzed by one-way ANOVA with *post hoc* LSD test (*n* = 8–12 images per group); ^•^*P* < 0.05, ^••^*P* < 0.01, ^•••^*P* < 0.005, and ^••••^*P* < 0.001. Significant differences between aged-matched Tgαq*44 and FVB mice were tested using two-sided Student’s *t*-test; *P < 0.05, **P < 0.01, ***P < 0.005, and ****P < 0.001.

Representative microphotograph of a specific, robust immunofluorescent staining of DMPO nitrone adducts in whole-heart cross-sections from 16-month-old Tgαq*44 mice with the use of anti-DMPO antibody is shown in Figure 1A. The signal was absent or very weak in age-matched FVB mice. High level of DMPO nitrone adducts in the heart apex from 16-month-old Tgαq*44 as compared with age-matched FVB was confirmed by Western Blot analysis (Figures 1B,C). Using immunofluorescent staining of DMPO nitrone adducts, progression of oxidative stress was quantified in 8- to 16-month-old Tgαq*44 mice as compared with age-matched FVB mice (Figure 2A). For 8-month-old mice, the fluorescent signal of DMPO nitrone adducts quantified collectively for the various areas of the hearts of Tgαq*44 and FVB was similar, but increased gradually with age (Figure 2B), attaining the significant difference between Tgαq*44 groups at the age of 10 months (••) and compared with FVB at the age of 12 months (*), with a profound amplification in 16-month-old Tgαq*44 mice. When the left and right ventricles, septum, and papillary muscles were analyzed independently, a moderate, yet statistically significant increase of DMPO-specific fluorescence, was also appreciated in 10-month-old Tgαq*44 mice in the left and right ventricles (Figures 3A,B), and in 12-month-old Tgαq*44 mice in the septum (Figure 3C); however, the papillary muscles (Figure 3D) became affected at the very late stage of HF (16-month-old Tgαq*44 mice).

### Oxidative Modifications in the Endothelium of Large- and Microcoronary Vessels

**Figure 4:**
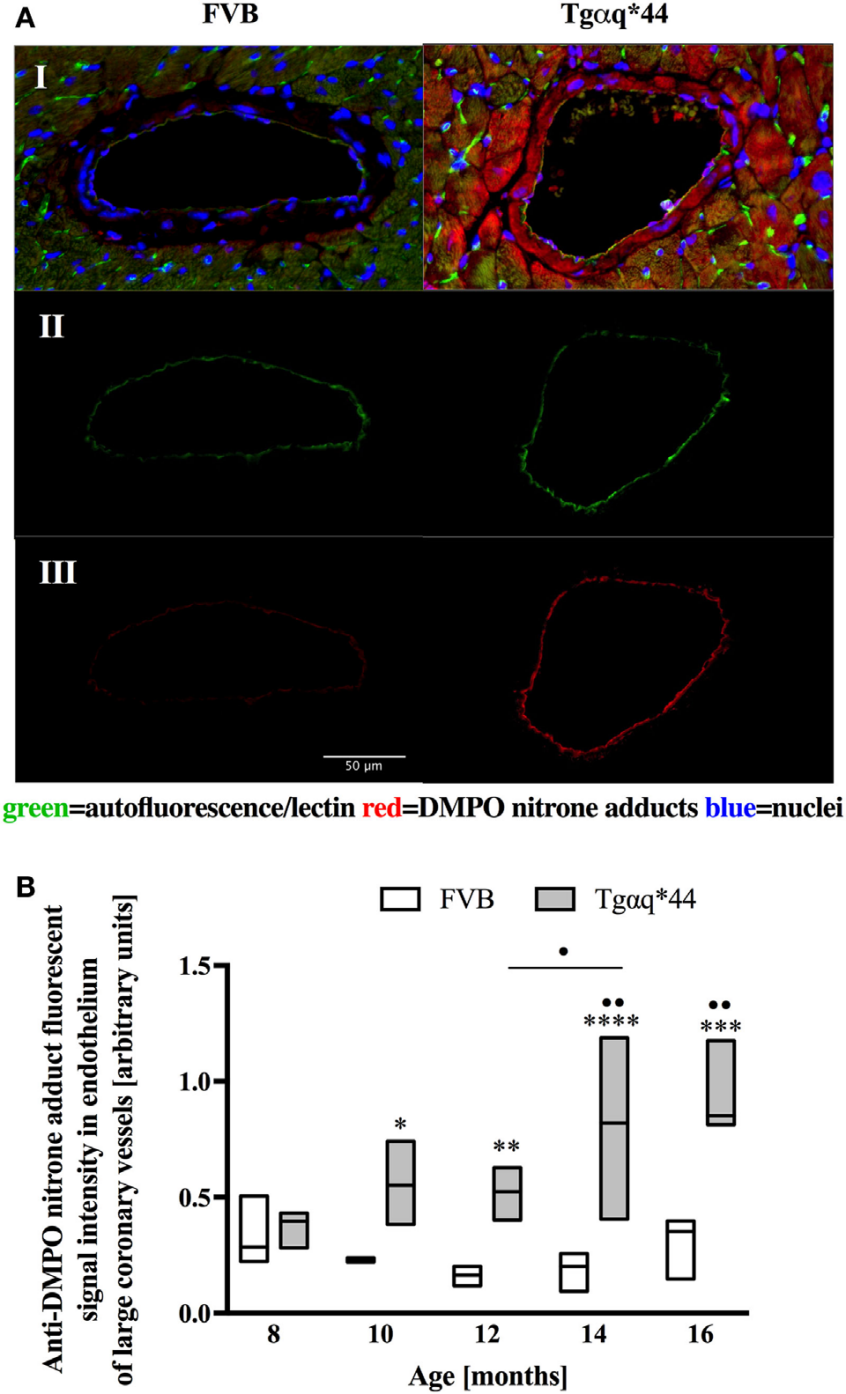
Oxidative modifications analyzed using immunohistochemical detection of DMPO nitrone adducts in the endothelium of large coronary vessels. **(A)** Segmentation steps for representative images of vessels in the left ventricle of 14-month-old mice (image size 250 μm × 150 μm); I: initial, triple-strained fluorescent images; II: endothelium segmentation based on lectin-positive staining of the vessel wall; III: DMPO nitrone adduct fluorescent signal specific to the endothelium area. **(B)** Quantification of the endothelium-specific oxidative modification (see Section “Materials and Methods” for details). Data presented as median ± IQR. Statistical significance was assessed using the two-way ANOVA, with *post hoc* LSD’s test between groups. *P < 0.05, **P < 0.01, ***P < 0.005, and ****P < 0.001 denote significant differences in aged-matched groups, while ^•^*P* < 0.05 and ^••^*P* < 0.01 between Tgαq*44 groups compared with 8-month-old Tgαq*44 group and 12- to 14-month-old groups. *N* = 3–6 mice/group, *n* = 5–6 vessels/mice.

Fluorescent detection of DMPO nitrone adducts, coupled with lectin co-staining, allowed for determination of oxidative modifications localized in the endothelium within the heart sections analyzed. Following a manual selection of large vessels from the left ventricle [vessel radius, expresses as median (Q1–Q3) was 42.9 (27.5–58.4) μm] for each mice, a lectin-positive area was automatically segmented and DMPO-specific fluorescence analyzed (Figure 4A). The general tendency was similar to that seen within the cardiomyocytes, with significant elevation of DMPO-specific fluorescence in 10-month-old Tgαq*44 mice, with the sharpest further increase seen between 12- and 14-month-old Tgαq*44 mice (Figure 4B).

**Figure 5:**
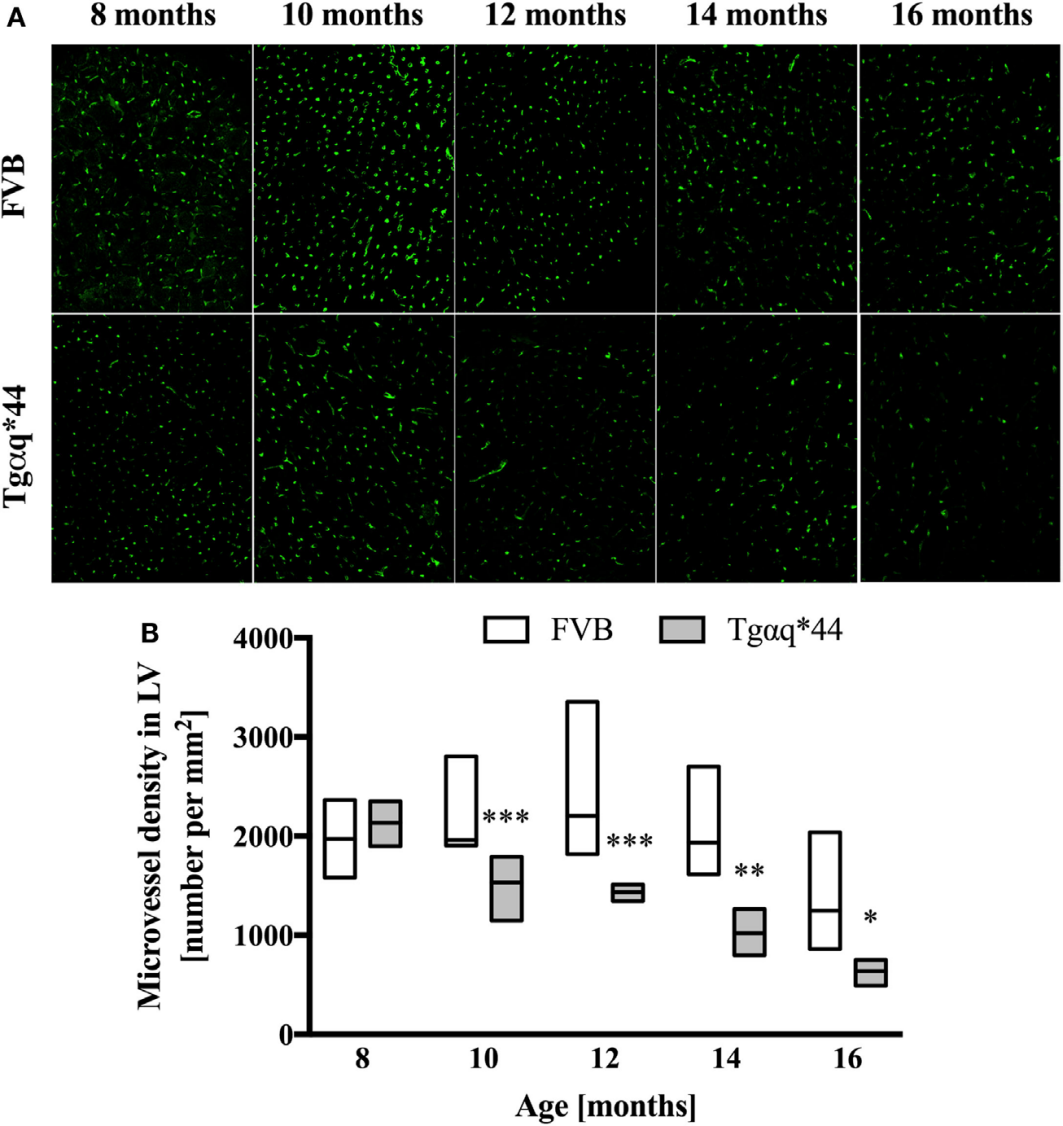
Microvessel density in the heart of Tgαq*44 and FVB mice. **(A)** Representative images of microvessels in the left ventricle stained using lectin from 8- to 16-month-old Tgαq*44 and FVB mice. **(B)** The number of microvessels per square millimeters, quantified using automated morphological manipulations of images on the thresholded FITC channel, as described in Section “Materials and Methods.” Data shown as median ± IQR. Statistical significance was assessed using two-way ANOVA, with *post hoc* LSD’s test between groups. *P < 0.05, **P < 0.01, and ***P < 0.005 denote significant differences in aged-matched groups. Furthermore, one-way ANOVA within Tgαq*44 mice showed a strong linear trend (*P* < 0.001) for decrease in microvessel density. *n* = 4–9 slices per group.

**Figure 6:**
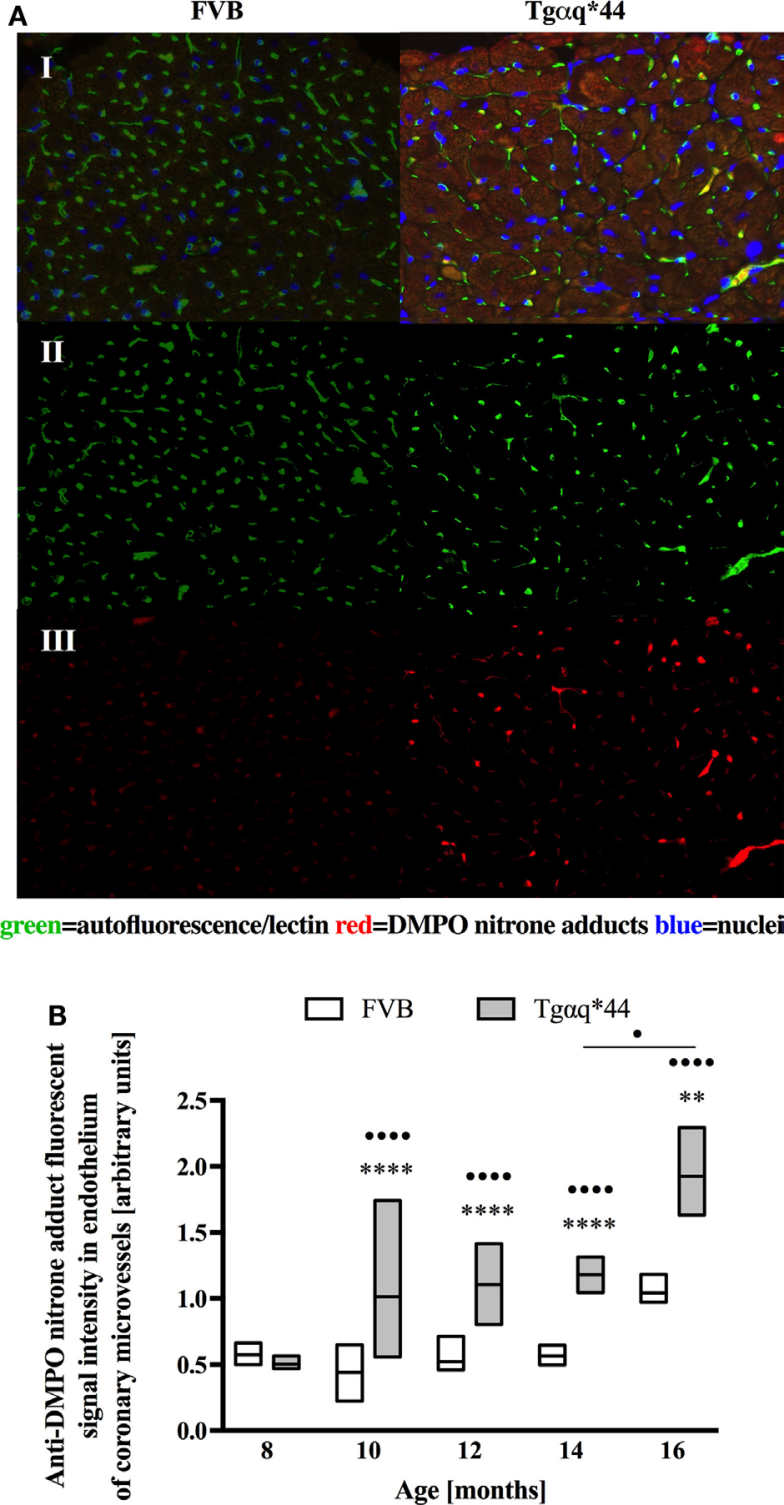
Oxidative modifications analyzed using immunohistochemical detection of DMPO nitrone adducts within the coronary microvessels in Tgαq*44 and FVB mice. **(A)** Microvessel-specific DMPO signal pre-processing steps for a representative pair of Tgαq*44 and FVB left ventricle free wall images of 12-month-old mice; I: initial, triple-strained fluorescent images; II: microvessel segmentation based on lectin-positive staining; III: DMPO nitrone adduct fluorescent signal specific to the microvessels, **(B)** quantification of the microvessel-specific fluorescence. Results presented as median ± IQR. Statistical significance was assessed using the ANOVA Kruskal–Wallis test, with *post hoc* Dunn’s test between groups. **P < 0.01 and ****P < 0.001 denote significant differences in aged-matched groups, while ^•^*P* < 0.05 and ^••••^*P* < 0.001 between Tgαq*44 groups compared with 8-month-old Tgαq*44 group and between 14- and 16-month-old Tgαq*44 mice. *N* = 4–6 mice/group, *n* ≥ 5 fields/mice.

Lectin-positive staining was also used to quantify the changes in the left ventricle microvasculature. There was a progressive deterioration of microvessel density in the Tgαq*44 mice, which begun at the age of 10 months and led to the loss of roughly 50% of lectin-positive microvessels in 16-month-old Tgαq*44 mice (Figure 5). Gradual loss of microvessels was also detected in the 16-month-old FVB mice strain, albeit these changes were not significant. Using lectin-positive staining, the DMPO nitrone adduct fluorescence within the microvessel endothelium was segmented (Figure 6A) and was clearly increased in Tgαq*44 mice as compared with FVB mice, starting at the age of 10 months, with a sharp increase for 16-month-old Tgαq*44 mice (Figure 6B).

### Superoxide Anion Production in the Heart

**Figure 7:**
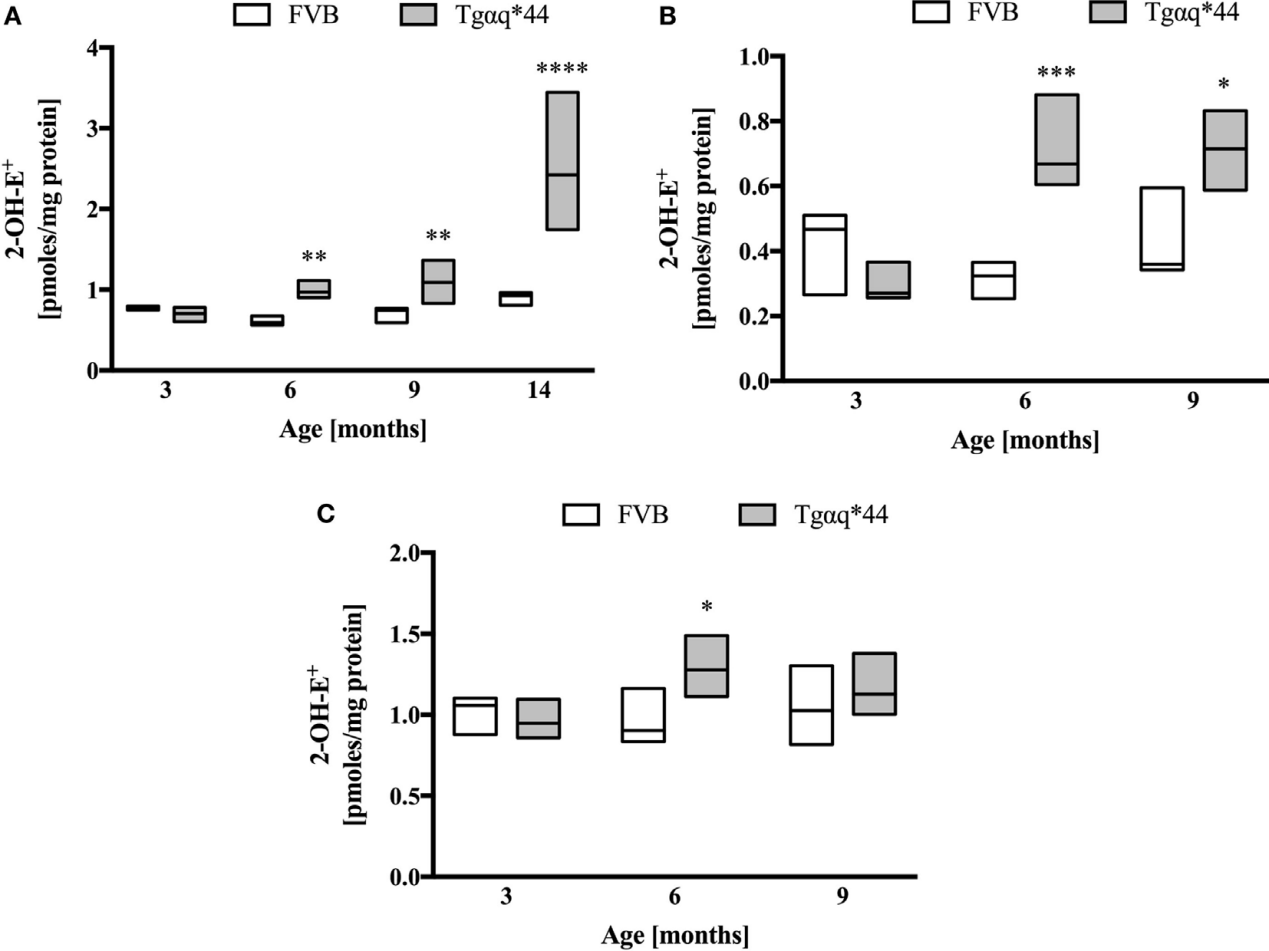
Superoxide production in heart homogenates of Tgαq*44 vs FVB mice. **(A)** Whole heart, **(B)** left ventricle and septum, and **(C)** right ventricle. Tissue samples were incubated with dihydroethidium, followed by superoxide-specific 2-hydroxyethiudium (2-OH-E+) detection using high performance liquid chromatography, as described in Section “Materials and Methods.” Data represent median ± IQR. Results presented as median ± IQR. Statistical significance in panel **(A)** for aged-matched groups was assessed using the non-parametric Mann–Whitney *U* test, *N* = 5–6 hearts (3- to 9-month-old groups) and *N* = 8–11 (14-month-old group). In panels **(B,C)**, statistical significance in aged-matched groups was assessed using the non-parametric Mann–Whitney test (for 3-month-old mice) or unpaired two-tailed *t*-test (all other), *N* = 5 hearts per group; *P < 0.05, **P < 0.01, and ***P < 0.005.

Heart homogenates were used to assess superoxide *ex vivo* using the HPLC-based detection of 2-hydroxyethidium (Figures 7A–C). When the entire heart was homogenized (Figure 7A), increased 2-hydroxyethidium levels in the heart was seen in Tgαq*44 mice at the age of 6–9 months, with a dramatic increase for 14-month-old Tgαq*44 mice. As shown in Figures 7B,C, in the left ventricle (isolated along with the septum) from 6- to 9-month-old Tgαq*44 mice 2-hydroxyethidium level increased significantly (Figure 7B), whereas in the right ventricle, despite apparently higher superoxide levels, there was only a modest increase in 6-month-old, but not in 9-month-old Tgαq*44 mice (Figure 7C).

### Activity of Antioxidant Enzymes and Redox State in the Heart

**Figure 8:**
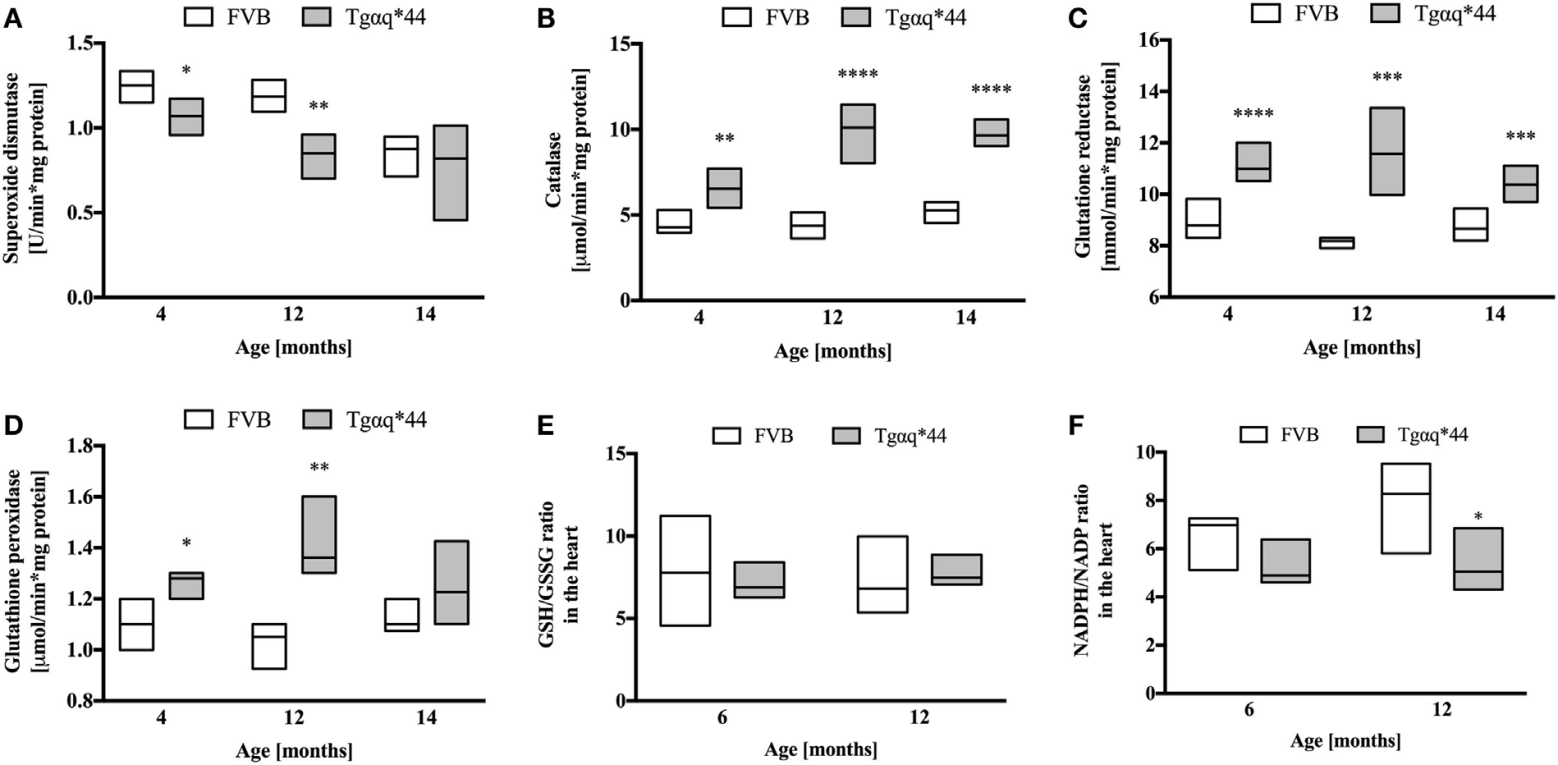
Levels of endogenous antioxidant enzymes activity and redox state in the hearts of Tgαq*44 vs FVB mice. **(A)** Superoxide dismutase, **(B)** catalase, **(C)** glutathione reductase, **(D)** glutathione peroxidase (GPx), **(E)** GSH/GSSG ratio, and **(F)** NADPH/NADP ratio. Data represent median ± IQR, *N* = 4–8 mice/group. Statistical significance between aged-matched groups was assessed using an unpaired two-tailed *t*-test, except data for GPx and 4-month data for catalase, where the non-parametric Mann–Whitney *U* test was used; *P < 0.05, **P < 0.01, ***P < 0.005, and ****P < 0.001.

Superoxide dismutase activity in Tgαq*44 mice was lower than in FVB controls, at a very early stage of HF development (at the age of 4 months) and further decreased in 12-month-old Tgαq*44 mice (Figure 8A). Interestingly, SOD activity also declined with age for the FVB controls and was comparable in 14-month-old Tgαq*44 and FVB mice. Other antioxidant enzymes studied (Figures 8B–D) exhibited an elevated activity in the Tgαq*44, when compared with aged-matched controls. This was evident and significant for catalase (Figure 8B), GR (Figure 8C) and GPx (Figure 8D). There were no significant differences in reduced glutathione levels in Tgαq*44 vs FVB mice [in µmol/g tissue for 4-month-old: 0.92 (0.86–0.97) vs 0.86 (0.82–0.89); 12-month-old: 0.99 (0.88–1.09) vs 0.9 (0.86–0.98) and 14-month-old: 0.95 (0.86–1.06) vs 0.92 (0.81–1.01)]. Furthermore, the ratio of GSH/GSSG remained unchanged at the early (6-month-old) and late (12-month-old) stages of HF development in these mice (Figure 8E); however, the NADPH/NADP ratio was decreased in Tgαq*44 vs FVB mice, significantly for 12-month-old mice (Figure 8F), due to significantly lower NADPH content in the hearts of Tgαq*44 vs FVB [in nmol/mg tissue for 6-month old: 13.7 (11.7–17.2) vs 21.3 (17.2–26.4) and 12-month old: 17.6 (14.3–18.8) vs 30.9 (21.9–35.6)].

## Discussion

In this study, we applied, to the best of our knowledge for the first time, DMPO IST-based method to detect *in vivo* oxidative modifications in cardiomyocytes and coronary endothelium of large- and microvessels in a mouse HF model. We provided evidence that in Tgαq*44 mice with slowly developing HF, resembling the progression of HF in humans on a molecular, morphological, and functional levels (4, 50, 54–57, 69), increased production of superoxide resulted in the development of oxidative modifications that occurred not only in cardiomyocytes, but simultaneously in the coronary endothelium at the transition phase of HF, before the end-stage disease. Altogether, these results underscore the important role of coronary endothelial dysfunction in the progression of HF, in a model driven by a cardiomyocyte-specific overexpression of Gαq* protein, whereby coronary endothelial function is initially preserved (56).

**Figure 9:**
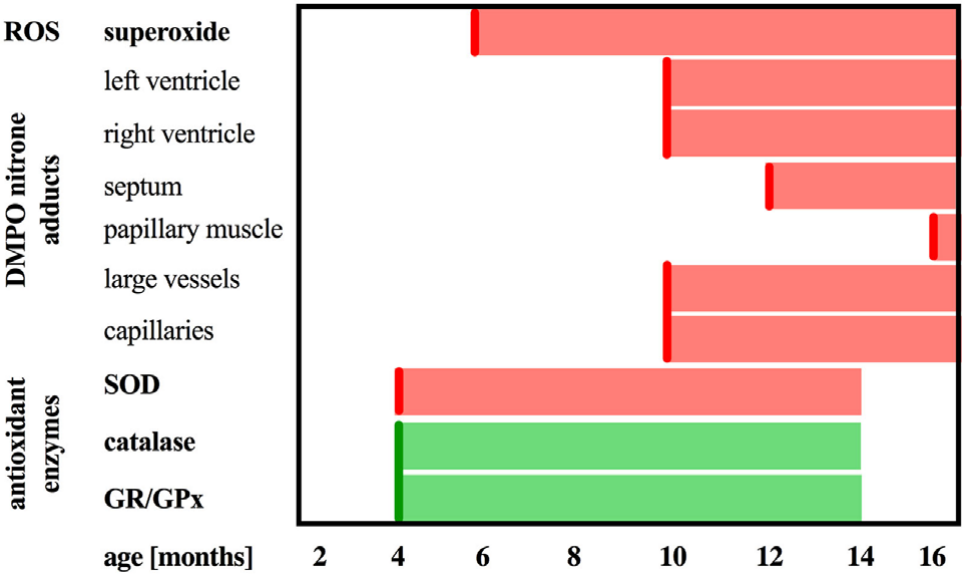
Summary of DMPO immuno-spin trapping results in relation to reactive oxygen species generation and antioxidant activity in Tgαq*44 mice. Scheme representing the sequence of events in terms of upregulation of catalase and glutathione reductase and peroxidase (GR/GPx), downregulation of superoxide dismutase (SOD) activity, increased superoxide production in the heart, followed by oxidative modifications in the heart in the relation to the age of Tgαq*44 mice.

Previous work identified three distinct phases of HF progression in Tgαq*44 mice; early (subtle diastolic perturbations at 6 months of age), transition (decreased basal cardiac function, with preserved cardiac reserve beginning at 8 months of age), and end-stage (impaired global cardiac performance and cardiac reserve starting at 12 months of age) (54). As summarized in Figure 9, in this work, we demonstrated that superoxide production in the heart was significantly increased in 6-month-old Tgαq*44 mice, which was accompanied by a compensatory activation of antioxidative mechanisms in the myocardium of Tgαq*44 mice, namely upregulation of catalase (CAT), glutathione reductase (GR), and glutathione peroxidase (GPx), opposed to superoxide dismutase (SOD) which was downregulated. Prior studies of oxidative stress in this animal model showed increased NADPH-oxidase dependent superoxide production using lucigenin assay in 2- to 4-month-old Tgαq*44 mice, which further progressed (56); however, changes in antioxidant systems were not as yet characterized. In this work, cardiac superoxide production was unaltered in 3-month-old Tgαq*44 mice, whereas the 14-month-old group showed a substantial increase (Figure 7A), with assay differences possibly explaining for the discrepancy in results for young mice between current and previous studies (56). Our results are compatible with the regulatory role of increased reactive oxygen species production in cardiomyocyte hypertrophy response and activation of fetal phenotype, which occurred quite early in Tgαq*44 mice, as evidenced by the activation of hypertrophic genes (ANP, BNP, and MHC-β), cardiomyocyte hypertrophy, fibrosis in 4-month-old Tgαq*44 mice (50, 51). On the other hand, mitochondrial dysfunction in cardiomyocytes using EPR detection of semiquinones content and Fe-S clusters (4), was identified in 10-month-old Tgαq*44 mice.

Here, we demonstrated that in Tgαq*44 hearts antioxidant systems are upregulated. Elevated activity of catalase (Figure 8B) is especially interesting, since cardiac-specific overexpression of catalase has been shown to abolish oxidative stress and prevent the progression to overt HF in an alternative Gαq-overexpressing transgenic mouse model (70). On the other hand, Nox4-derived hydrogen peroxide both in cardiomyocytes and endothelial cells was shown to mediate protection against pressure overload cardiac remodeling (71). Our analysis showed that increased activity of CAT was associated with elevated activity of GPx, which overexpression in mice was previously shown to prevent left ventricular failure after myocardial infarction (72). Since both enzymes are involved in hydrogen peroxide metabolism, it might well be that their upregulation protect the failing heart synergistically. GR activity was also enhanced in Tgαq*44 mice, which might have contributed to NADPH depletion and a decreased NADPH/NADP ratio for 12-month-old Tgαq*44 mice. On the other hand, increased GR activity could at least partially explain the preservation of GSH cardiac pool and GSH/GSSG ratio, despite increased ROS production.

In contrast to early increase in ROS production and early activation of antioxidant systems in Tgαq*44 mice, fluorescent detection of DMPO nitrone adducts in the whole heart showed a statistically significant increase in Tgαq*44 mice compared with aged-matched FVB mice starting at the age of 12 months, with an age-dependent progression (Figure 2) and a sharp increase in signal for the 16-month-old Tgαq*44 mice. Comparable trends were appreciated when the left or right ventricle and septum regions of the heart were analyzed independently, with only exception being the papillary muscle, where oxidative modifications developed only for the 16-month-old Tgαq*44 mice (Figure 3). The specificity of fluorescent IST analysis was confirmed using Western Blot detection of DMPO nitrone adducts in heart apex homogenates, with a nearly sixfold intensity increase in DMPO nitrone adducts in the myocardium of Tgαq*44 mice, with a semi-quantitative analysis (Figure 1C) of the protein band at roughly 150 kDa. This particular band might be related to oxidatively modified oxygen-regulated protein 150, a chaperonin known to be expressed in tissues undergoing hypoxic or endoplasmic reticulum stress (73), involved in VEGF transport (74). However, here we did not analyze the origin of the 150 kDa band seen in DMPO-specific Western Blot that might be determined by mass spectrometry (75). We used this band as a representative for the analysis and quantification of the oxidative modification process. Obviously there are a number of protein modifications reported to form DMPO nitrone adducts that can be identified, for example tyrosine nitration of carboxypeptidase B1 (76), Cys and tyrosine superoxide-specific modifications of NADH dehydrogenase (77) or superoxide-dependent succinate ubiquinone reductase (SQR)-derived protein radical (78). It is worth adding that the extent of protein radicals reacting with DMPO is counterbalanced in part by intrinsic reactions with GSH (79, 80) (and ascorbate, molecular oxygen lipid, or other radicals), being a potential source of underestimation of oxidative modifications detected by IST (41).

In this work, we analyzed and quantified oxidative modifications not only in cardiomyocytes but also in coronary endothelium in Tgαq*44 hearts based on co-staining of the left ventricle with anti-DMPO and lectin antibodies that enabled the segmentation and quantification of DMPO nitrone adducts specific to the endothelium of large coronaries and microvessels. Literature describes the use of CD31 (81) or various lectins (82–84) for imaging of the capillary bed. In our experience, the lectin antibody (see Materials and Methods) was better suited to stain the capillary vessels within the left ventricle specimens, as reported previously (85). Compared with FVB controls, Tgαq*44 mice had more oxidative modifications found in the endothelium from the age of 10 months onward, regardless of whether large vessels (Figure 4) or capillaries (Figure 6) were analyzed. The most pronounced increase in oxidative modifications to the endothelium of large coronaries and microvessels was found in the end-stage of HF, at 12–16 months of age. Using the lectin-positive staining, we were also able to quantify the capillary density in the left ventricle (Figure 5). For the youngest mice group, both Tgαq*44 and control mice exhibit start with around 2,000 capillaries/mm^2^. Already at 10 months of age, Tgαq*44 mice display a significant decrease in capillary density, which progressed to below 1,000 capillaries/mm^2^ for 16-month-old Tgαq*44 mice. The loss of capillaries in this HF model was not due to cardiomyocyte hypertrophy, as normalization of number of capillaries to cardiomyocyte length, width or their volume, did also confirm capillary loss (data not shown). Interestingly, as shown by Tyrankiewicz et al. (54), a prominent ACE/Ang II pathway activation was present at the phase of decompensated HF suggesting that ACE/Ang II pathway could be involved in development of coronary endothelial damage (86). Obviously, number of other mechanisms could be involved in coronary capillary loss and their dysfunction in HF in Tgαq*44 mice, and they were not studied here.

Various *in vivo* studies using DMPO-based IST are described in literature; however, this work is the first to implement and extend the IST methodology in an *in vivo* protracted HF model, with an additional emphasis on cellular localization and recognition of the progression of oxidative modifications in the cardiomyocytes and coronary endothelium in relationship with functional deteriorations of the cardiac function in this model as reported previously (54). Indeed, most previous work studied the effects of acute and lethal oxidative interventions, such as acetone-induced ketosis (87), lipopolysaccharide-induced systemic inflammation (76), or rat liver ischemia/reperfusion injury model (88). A more recent report described free radical formation in high fat diet in mice and in monocrotaline-induced pulmonary hypertension, and right HF rat model (43), demonstrated considerably more DMPO nitrone adducts in both diseases; however, the authors did analyze only the late stage of the disease progression. In contrast, in this work, we provided evidence that IST can reveal progressive nature of oxidative modifications along the extended time period of the progression of HF in Tgαq*44 mice (52).

## Conclusion

In this work, we characterized oxidative modifications in HF in Tgαq*44 mice. The increased abundance of DMPO nitrone adducts identified in the myocardium of Tgαq*44 mice occurred at the stage of impairment of basal systolic and diastolic cardiac performance, and diminished capillarization of Tgαq*44 hearts, suggesting that the presence of DMPO nitrone adducts in cardiomyocytes, and in coronary endothelium may reflect a stage of a significant cardiac and cardiac capillaries damage of a failing heart. Given the fact that HF in Tgαq*44 mice is initiated by a cardiomyocyte-specific alteration, development of oxidative modifications in parallel in cardiomyocytes and in the endothelium of large coronaries and capillaries suggests that a cardiomyocyte-derived mechanism is responsible for coronary capillaries damage and might contribute to the progression of HF in Tgαq*44 mice. The nature of this mechanism remains to be established.

## Ethics Statement

All experimental procedures were compliant with the Guide for the Care and Use of Laboratory Animals published by the U.S. National Institutes of Health (NIH Publication No. 85-23, revised 1996) and were approved by the Second Local Ethical Committee on Animal Testing at the Institute of Pharmacology PAN in Krakow, Poland (permit no. 15/2016).

## Author Contributions

Conceived and designed the study: BP and SC. Performed the study: BP, JC, TK, KK, and AZ. Analyzed the data: BP and JC. Drafted the manuscript: BP, JC, and SC. All the authors have corrected or have approved the final version of the manuscript.

## Conflict of Interest Statement

The authors declare that the research was conducted in the absence of any commercial or financial relationships that could be construed as a potential conflict of interest.
